# Impact of Cardiopulmonary Bypass on Respiratory Mucociliary Function in an Experimental Porcine Model

**DOI:** 10.1371/journal.pone.0135564

**Published:** 2015-08-19

**Authors:** Rodrigo Sánchez-Véliz, Maria José Carmona, Denise Aya Otsuki, Claudia Freitas, Anderson Benício, Elnara Marcia Negri, Luiz Marcelo Malbouisson

**Affiliations:** 1 Laboratory of Anesthesiology (LIM08), University of Sao Paulo School of Medicine, Sao Paulo, Brazil; 2 Cardiac Surgery Division, Heart Institute (InCor), University of Sao Paulo School of Medicine, Sao Paulo, Brazil; 3 Department of Pathology, Experimental Air Pollution Laboratory, University of Sao Paulo School of Medicine, Sao Paulo, Brazil; University of Bari, ITALY

## Abstract

**Background:**

The impact of cardiac surgery using cardiopulmonary bypass (CPB) on the respiratory mucociliary function is unknown. This study evaluated the effects of CPB and interruption of mechanical ventilation on the respiratory mucociliary system.

**Methods:**

Twenty-two pigs were randomly assigned to the control (n = 10) or CPB group (n = 12). After the induction of anesthesia, a tracheostomy was performed, and tracheal tissue samples were excised (T0) from both groups. All animals underwent thoracotomy. In the CPB group, an aorto-bicaval CPB was installed and maintained for 90 minutes. During the CPB, mechanical ventilation was interrupted, and the tracheal tube was disconnected. A second tracheal tissue sample was obtained 180 minutes after the tracheostomy (T180). Mucus samples were collected from the trachea using a bronchoscope at T0, T90 and T180. Ciliary beat frequency (CBF) and *in situ* mucociliary transport (MCT) were studied in *ex vivo* tracheal epithelium. Mucus viscosity (MV) was assessed using a cone-plate viscometer. Qualitative tracheal histological analysis was performed at T180 tissue samples.

**Results:**

CBF decreased in the CPB group (13.1 ± 1.9 Hz vs. 11.1 ± 2.1 Hz, p < 0.05) but not in the control group (13.1 ± 1 Hz vs. 13 ± 2.9 Hz). At T90, viscosity was increased in the CPB group compared to the control (p < 0.05). No significant differences were observed in *in situ* MCT. Tracheal histology in the CPB group showed areas of ciliated epithelium loss, submucosal edema and infiltration of inflammatory cells.

**Conclusion:**

CPB acutely contributed to alterations in tracheal mucocilliary function.

## Introduction

The use of cardiopulmonary bypass (CPB) during cardiac surgery has been consistently associated with the development of postoperative pulmonary dysfunction.[1, 2] However, little is known about the impact of CPB on the tracheal mucociliary transport function.

The mucociliary system is considered one of most important defense mechanisms of the respiratory tract [3] and its efficient activity depends on 1) the coordinated beating of the cilia lining the respiratory tract, which propels mucus to the oropharynx; 2) possessing mucus of an adequate quantity and viscosity onto which particles and organisms can adhere; and 3) adequate periciliary fluid composition to optimize mucociliary clearance and to provide a milieu in which airway antimicrobial agents are effective. [3–5]

Factors such as infections, airway manipulation and mechanical ventilation have been reported to interfere with the mucociliary clearance by destroying the cilia cells or altering mucus production and may lead to sputum retention, airway occlusion and ventilator-associated pneumonia.[6–9] Despite the fact that some of these mechanisms are present during cardiac surgery, no studies have investigated the impact of surgical intervention with CPB on mucociliary transport. The purpose of this study was to evaluate the impact of CPB on ciliary beat frequency, mucus viscosity and histological alterations in the lower respiratory system in a porcine model of cardiac surgery undergoing CPB.

## Methods

Ethical approval for this study (CAPPesq—protocol number 756/06) was provided by the Ethics committee for research projects at the São Paulo University School of Medicine, São Paulo, Brazil. All procedures were performed according to the recommendations of Brazilian Society of Laboratory Animal Science (SBCAL) guidelines for ethical animal research. Twenty two pigs (mixed Large White and Landrace breeds) with weight ranging between 33 and 47 kg were studied. All animals came from a high-standard farm. All animals were fasted overnight before the experiment but given free access to water. On the morning of the experiment, all animals were examined by a veterinarian to certify their health status and exclude the presence of diseases, such as respiratory infections, before transport to the laboratory.

### Instrumentation

All animals received 5 mg/kg ketamine and 0.25 mg/kg midazolam intramuscularly at the moment of arrival in the laboratory. A Teflon cannula was inserted in the marginal ear vein, and general anesthesia was induced with 3–5 mg/kg propofol and maintained with 1.2% isoflurane, 3 μg/kg/h fentanyl and 5 μg/kg/h pancuronium. A cuffed endotracheal tube (inner diameter of 6.5 mm) was inserted, and mechanical ventilation was initiated (Primus, Dräger, Lubeck, Germany) with tidal volume set at 10 mg/kg, a respiratory rate of 15 breaths per minute, positive end-expiratory pressure (PEEP) of 5 cmH_2_O and inspired oxygen fraction of 0.6. Inspiratory time was adjusted to 33% of total respiratory cycle time, and an inspiratory pause was set to 10% of inspiratory time. Lactated Ringer’s solution was continuously infused at 10 mL/kg/h during the experiment. Heart rate, continuous ECG, esophageal temperature and pulse oximetry were monitored in all animals with a multiparametric monitor (IntelliVue MP40, Philips Medical Systems, Netherlands). End-tidal CO_2_, inspiratory oxygen and isoflurane concentrations were monitored using a gas analyzer (Criticare Systems INC, WI, USA). Peak, plateau and end-expiratory pressure were obtained directly from the ventilator after automatic calibration of the pneumotachograph. A heat and moisture exchanger (HME) (Humid-Vent Compact-S, Gibeck, Sweden) with a dead space of 38 mL was placed between the Y piece connecting the respirator circuit and the tracheal tube to maintain constant temperature and humidity throughout the study.

An 18-gauge catheter was inserted in the femoral artery via cut-down for pressure measurements and blood sampling. A thermodilution pulmonary artery catheter (catheter model 131 HF7 –Baxter Healthcare Corporation, CA, USA) was introduced through the right internal jugular vein, and its correct position was confirmed by pulmonary artery pressure curve dampening when the catheter tip balloon was insufflated. The cardiac index (CI) was calculated to normalize the data for body surface area in m2 by using a conversion factor appropriate for pigs (k·body weight ^2/3^, where k = 0.09). [10]

After anesthesia induction and the monitoring procedures, a tracheostomy was performed to facilitate tracheal mucus sampling and to excise a sample of tracheal tissue. A lung expansion maneuver was performed using a sustained airway pressure of 30 cmH_2_O for 15 seconds to reverse atelectasis related to anesthesia induction and airway manipulation before baseline measurements.[11]

### Sternotomy and cardiopulmonary bypass

A median sternotomy was performed, and the pericardium and both pleurae were opened in all animals. In the control group, the thoracotomy was covered with sterile sheets to avoid dehydration and hypothermia. In the CPB group, 500 IU/kg heparin was administered intravenously after sternotomy. Vascular cannulas were selected according to the size of the anatomic structure and were inserted both through the right atrial appendage and into the ascending aorta. The extracorporeal circuit and membrane oxygenator (Braile Biomédica, São José do Rio Preto, Brazil) were primed with 1500 ml lactated Ringer’s solution, 1 g/kg mannitol and 10000 IU heparin. After cardiopulmonary bypass was initiated, ventilation was stopped and esophageal temperature was maintained at 38°C. Mechanical ventilation was restarted before weaning of the CPB, and anticoagulation was reversed in the CPB group with protamine sulfate (1 mg for each 100 IU heparin) after removal of the vascular cannulas. No vasoactive drugs were used during CPB weaning. After the end of the experiment, the animals were euthanized with 2.5 mEq/kg potassium chloride using guidelines from the Report of the American Veterinary Medical Association Panel on Euthanasia. [12]

### Experimental design

The animals were randomly assigned into a control group (n = 10), in which mechanical ventilation and sternotomy were performed without CPB installation for a period of 180 minutes, or the CPB group (n = 12), in which CPB was performed for 90 minutes without mechanical ventilation, followed by an observation period of 90 minutes after CPB weaning. The random sequence of group allocation was obtained using the Stata 11 statistical software (StataCorp, College Station, TX, USA), and the allocation of each animal was enclosed in a brown numbered envelope and revealed at the start of each experiment.

Tracheal mucus, arterial and venous blood samples, standard hemodynamic measurements and respiratory mechanics were obtained at 3 time points: T0, after tracheostomy; T90, 90 minutes after the baseline in the Control group and at the end of CPB in the CPB group; and T180, 180 minutes after baseline. Inspiratory peak and mean end-inspiratory pressures were directly measured from the proximal ventilator transducer. The compliance of the respiratory system (Crs), PaO_2_/FiO_2_ ratio, intrapulmonary shunt and alveolar-arterial oxygen gradient were calculated using standard formulas.

A 0.5 cm^2^ sample of tracheal tissue was excised from the cephalic portion of the tracheal incision during tracheotomy after initial monitoring (T0). At the end of the experiment (T180), a second tracheal sample was excised from the caudal edge of the tracheostomy incision, which was not previously manipulated. All samples were immediately submitted to analysis.

### Evaluation of tracheal epithelium behavior

#### *Ex vivo* ciliary beat frequency (CBF)

Tracheal CBF was measured using a light microscope (Olympus BX 50, Tokyo, Japan) connected to a video camera (Sony -3CCD-color video camera) and a video monitor. A stroboscope (Machine Vision Strobe, Cedar Hurst, NY) was placed in front of the tracheal tissue fragment and emitted light flashes at a rate varying between 0 and 30 Hz, based on synchronization with the cilia movements. [13]

#### *In situ* mucociliary transport (MCT)

After CBF was evaluated, the same tracheal tissue fragment was used to monitor *in situ* MCT by the direct observation of charcoal particles in saline solution mixed with common talc particles using a 100X microscope. The progression of the solution drop across the trachea was timed and expressed as millimeters per second. [14]

#### Qualitative histological analysis

After the last measurement of MCT and CBF, an epithelial sample of the trachea was collected and fixed with 4% formalin-buffered solution for routine histological processing. Three animals were analyzed from each group for qualitative analysis of the tissue sample obtained at T180. These fragments were dehydrated in an ethanol gradient (70° to 100°), cleared with xylol and blocked in paraffin. Five-micrometer sections were obtained using a Leica RM2065 microtome. The sections were deparaffinized with xylol, hydrated in an ethanol gradient (100° to 70°) and dyed for 2 minutes with Harris’s hematoxylin. The sections were then washed in running water and counterdyed with eosin for 15 minutes. They were then washed in running water, dehydrated in an ethanol gradient (95° to 100°), cleared with xylol and mounted with laminule and entellan to analyze the histopathological alterations. The samples were qualitatively analyzed with an optical microscope by a pathologist blind to the group allocation.

#### Evaluation of respiratory mucus viscosity (MV)

Mucus samples were collected from the bronchial passages of the right principal superior bronchi using a bronchoscope (PENTAX FB-I5bs, JAPAN) and a protected brush at T0, T90 and T180. All mucus samples were placed into labeled tubes with mineral oil to prevent dehydration, and the samples were stored at -70°C until analysis.

Viscosity was measured using a Brookfield LVDV-II+Pro cone and plate digital viscometer with CP-40 spindle (Brookfield; Middleboro, MA) adapted to Wingathe software. The measurements were carried out at 21°C using a water bath with temperature controller. Viscosity measured at 100 rpm was used to compare the groups because this shear rate allowed us to measure the mucus viscosity of all of the samples without exceeding the torque (%) limitations of the instrument. Viscosity is expressed as pascals X seconds X 10^−3^ (mPa/s).

### Statistical Analysis

All data are presented as the means ± standard deviations unless otherwise specified. Weight and body surface area of the animals were compared between the groups using an unpaired Student’s *t*-test. Gender ratios in the groups were compared by Chi-square test. Hemodynamics, respiratory mechanics, gasometrics and data related to the assessment of respiratory mucociliary function were analyzed using two-way repeated-measures analysis of variance followed by the Student-Newman-Keuls test when indicated. Data from mucus viscosity measurements were compared between groups at each time point using the Mann-Whitney test due to the non-parametrical distribution of the data. P values below 0.05 were considered significant.

## Results

As shown in Table 1, there were no differences in the basic characteristics of the groups. Table 2 summarizes the hemodynamic and mechanical respiratory behavior during in this study. No differences were observed in the hemodynamics between groups. Cardiopulmonary bypass did not induce cardiovascular alterations, and weaning occurred without the need for vasoactive drugs. The CPB group had a non-statistically significant reduction in Crs of 23%, from 46 ± 13 mL/cmH_2_O to 35 ± 8 mL/cmH_2_O after CPB weaning, which persisted until the end of the experiment. Peak and plateau pressures were similar in both groups throughout the study.

**Table 1:** Morphometric characteristics of the studied animals. Mean ± SD.

	Control (n = 10)	CPB (n = 12)	p value
Gender (M/F)	7/3	11/1	ns
Weight (kg)	39.4 ± 3.9	41.3 ± 2.8	ns
BSA (m^2^)	1.04 ± 0.09	1.07 ± 0.05	ns

Legend: BSA—body surface area; ns—non-significant.

**Table 2:** Hemodynamic and mechanical respiratory behavior of the control and CPB groups throughout the study.

	Group	T0	T90	T180	p value
HR (bpm)	CPB	98.4 ± 17.1	125.5 ± 16.4	121.4 ± 20.4	ns
	Control	109.4 ± 18.0	116.2 ± 18.1	123.6 ± 18.7	
MAP (mmHg)	CPB	65.9 ± 8.4	65.0 ± 13.1	60.0 ± 4.8	ns
	Control	69.8 ± 5.6	79.7 ± 9.2	76.2 ± 7.8	
MPAP (mmHg)	CPB	17.2 ± 2.3	17.2 ± 2.1	17.5 ± 1.7	ns
	Control	17.8 ± 1.6	19.1 ± 2.4	19.4 ± 4.3	
CVP (mmHg)	CPB	7.4 ± 1.7	7.4 ± 1.1	7.4 ± 1.2	ns
	Control	7.6 ± 2.2	7.5 ± 2.3	7.5 ± 2.1	
PAOP (mmHg)	CPB	10.0 ± 2.9	8.9 ± 3.5	9.3 ± 2.9	ns
	Control	11.0 ± 3.4	10.5 ± 2.3	11.0 ± 2.2	
CI (L.min^-1^.m^2^)	CPB	4.0 ± 0.7	3.6 ± 0.7	3.3 ± 0.4	ns
	Control	3.9 ± 0.8	4.3 ± 0.7	4.5 ± 0.9	
SVRI (din.s.cm^5.^m^-2^)	CPB	1179 ± 205	1308 ± 358	1312 ± 214	ns
	Control	1345 ± 367	1355 ± 180	1252 ± 218	
PVRI (din.s.cm^5.^m^-2^)	CPB	145 ± 77	192 ± 60	209 ± 59	ns
	Control	144 ± 57	159 ± 59	147 ± 57	
P_peak_ (cmH_2_O)	CPB	16.4 ± 1.7	19.3 ± 2.5	20.0 ± 2.4	ns
	Control	18.6 ± 2.9	19.4 ± 3.1	19.4 ± 3.3	
P_Plateau_ (cmH2O)	CPB	15.3 ± 2.2	18.5 ± 3.3	18.0 ± 1.3	ns
	Control	16.7 ± 3.2	17.7 ± 2.9	17.4 ± 3.6	
C_rs_ (m/cmH2O^-1^)	CPB	45.9 ± 13.3	35.3 ± 7.7	34.7 ± 4.8	ns
	Control	42.9 ± 7.0	46.0 ± 10.7	44.3 ± 11.3	

Legend: HR—heart rate; MAP—mean arterial pressure; MPAP—mean pulmonary arterial pressure; CVP—central venous pressure; PAOP—pulmonary artery occlusion pressure; CI—cardiac index; SVRI—systemic vascular resistance index; PVRI—pulmonary vascular resistance index; Ppeak—peak airway pressure; Plateau—plateau airway pressure; Crs—static compliance of the respiratory system; ns—non-significant

After 90 minutes, there was a 50% decrease in the PaO_2_/FiO_2_ ratio in the CPB group (from 428 ± 45 mmHg to 214 ± 96 mmHg, p < 0.05). At T180, PaO_2_/FiO_2_ ratio values increased compared to T90, however remaining lower than baseline (332 ± 112 mmHg, p < 0.05). Alveolar-arterial oxygen gradient and pulmonary shunt significantly increased at T90 in CPB group (from 95 ± 25 mmHg and 8.5 ± 2.3% to 223 ± 59 mmHg and 21.4 ± 9.8%, respectively, p < 0.05) and decreased at T180 but did not return to baseline levels. No significant alterations in gas exchange parameters were observed in the control group (Table 3). The core temperature, other blood gas and biologic parameters remained within the normal range and presented similar behaviors in both groups throughout the study. Supporting information can be found in S1 File.

**Table 3:** Oxygenation and metabolic variable behavior throughout the study.

	Group	T0	T90	T180	p value
Pao_2_/FiO_2_ (mmHg)	CPB	428 ± 4	214 ± 96* #	333 ± 111* † #	0.003
	Control	393 ± 8	438 ± 50	426 ± 58	
GA-aO_2_ (mmHg)	CPB	94.6 ± 25.0	223.5 ± 59.3* #	152.8 ± 68.4* † #	0.009
	Control	108.2 ± 27.9	96.8 ± 37.6	107.0 ± 44.4	
Shunt (%)	CPB	8.5 ± 2.3	21.4 ± 9.8* #	10.4 ± 6.9	0.091
	Control	10.0 ± 5.5	8.9 ± 4.4	9.0 ± 5.4	
PaCO_2_ (mmHg)	CPB	41.2 ± 2.6	44.9 ± 4.5	40.5 ± 3.9	ns
	Control	38.2 ± 5.1	38.8 ± 1.6	39.2 ± 3.9	
Arterial HCO_3_^-^ (mEq/L)	CPB	28.7 ± 1.8	26.2 ± 1.4	27.6 ± 2.0	ns
	Control	28.0 ± 1.7	28.3 ± 1.5	29.3 ± 1.6	
Base deficit (mEq/L)	CPB	-5.1 ± 1.6	-1.6 ± 1.0	-3.9 ± 2.0	ns
	Control	-3.9 ± 1.8	-4.6 ± 1.4	-5.4 ± 1.8	
pH	CPB	7.45 ± 0.03	7.38 ± 0.04	7.45 ± 0.04	ns
	Control	7.46 ± 0.05	7.37 ± 0.05	7.47 ± 0.05	
Hematocrit (%)	CPB	28.4 ± 3.5	26.5 ± 2.5	26.3 ± 4.1	ns
	Control	28.0 ± 1.7	27.8 ± 2.6	28.8 ± 1.7	
Esophageal temperature (°C)	CPB	37.9 ± 0.8	37.7 ± 0.6	38.1 ± 0.7	ns
	Control	37.9 ± 0.8	38.0 ± 0.6	38.0 ± 1.1	

Legend: Pao_2_/FiO_2_—Pao_2_/FiO_2_ ratio; GA-aO_2_ –alveolar—arterial oxygen pressure difference

* p value < 0.05 compared to T0

^#^ p value < 0.05 when the same time is compared to the control group

^†^ p value < 0.05 compared to T90; ns—non-significant

### Mucociliary function

**Fig 1:**
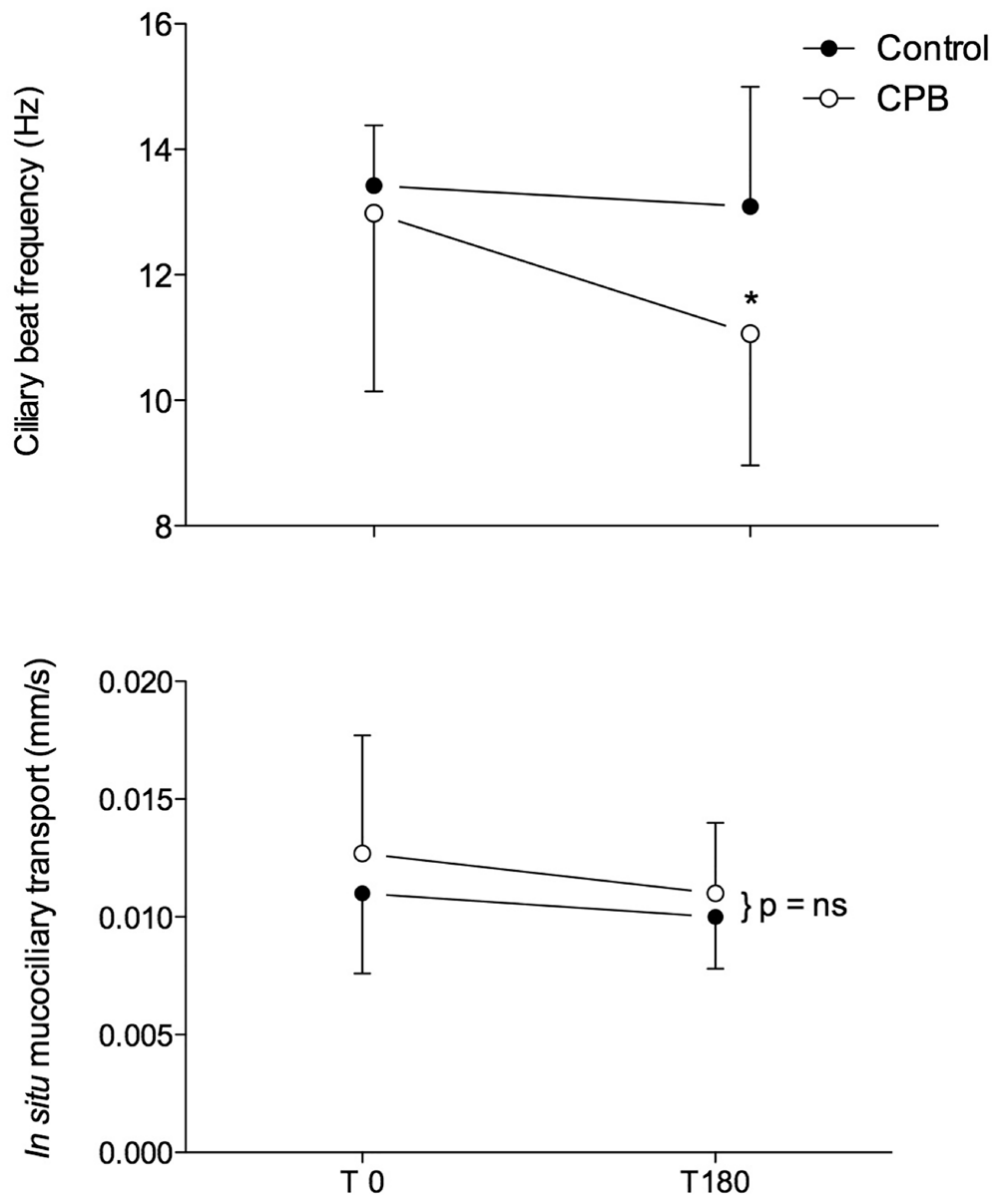
Behavior of ciliary beat frequency (upper panel) and *in situ* mucociliary transport (lower panel) in the control group (closed circles) and in the CPB group (open circles) throughout the study. * means different from baseline (p < 0.05). Data expressed as the means ± SD.

The CPB group had a significant reduction in CBF at T180 compared to T0 (T0: 13 ± 2.8; T180: 11.1 ± 2.1 Hz, p < 0.05). However, CBF did not change in the control group when T0 was compared to T180. No significant alterations were observed in *in situ* MCT (Fig 1). Supporting information can be found in S2 File

### Mucus viscosity

**Fig 2:**
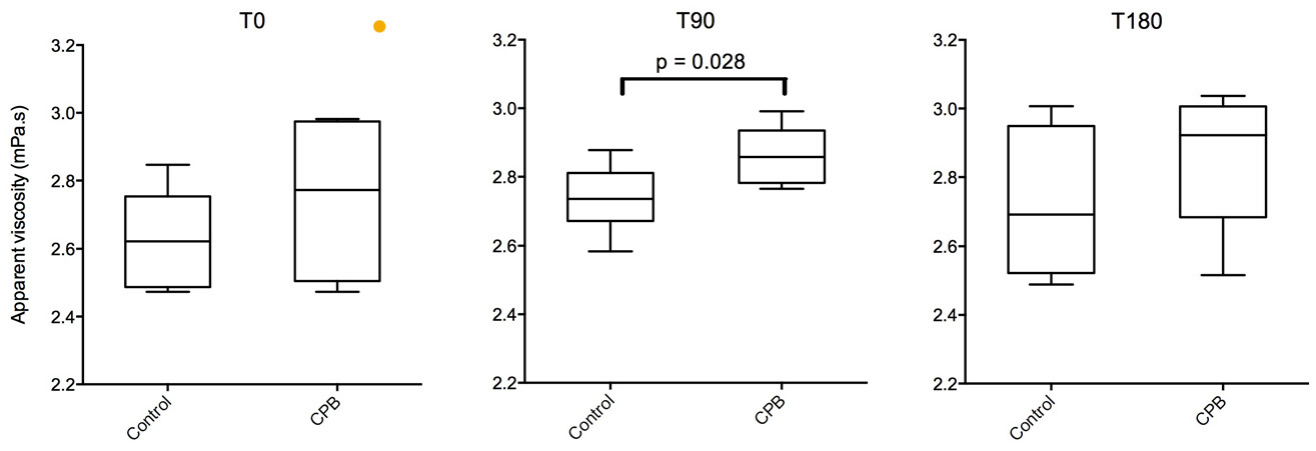
Apparent viscosity of respiratory mucus collected by bronchoscopy in the control and CPB groups at T0 (left panel), T90 (middle panel) and T180 (right panel). Box and whiskers represent the 25th–75th percentile and minimal–maximal values, respectively. The line in each box represents the median.

As shown in Fig 2, the apparent viscosity was reversibly increased at T90 in the CPB group and returned to basal values at T180 (S3 File).

### Tracheal histology

**Fig 3:**
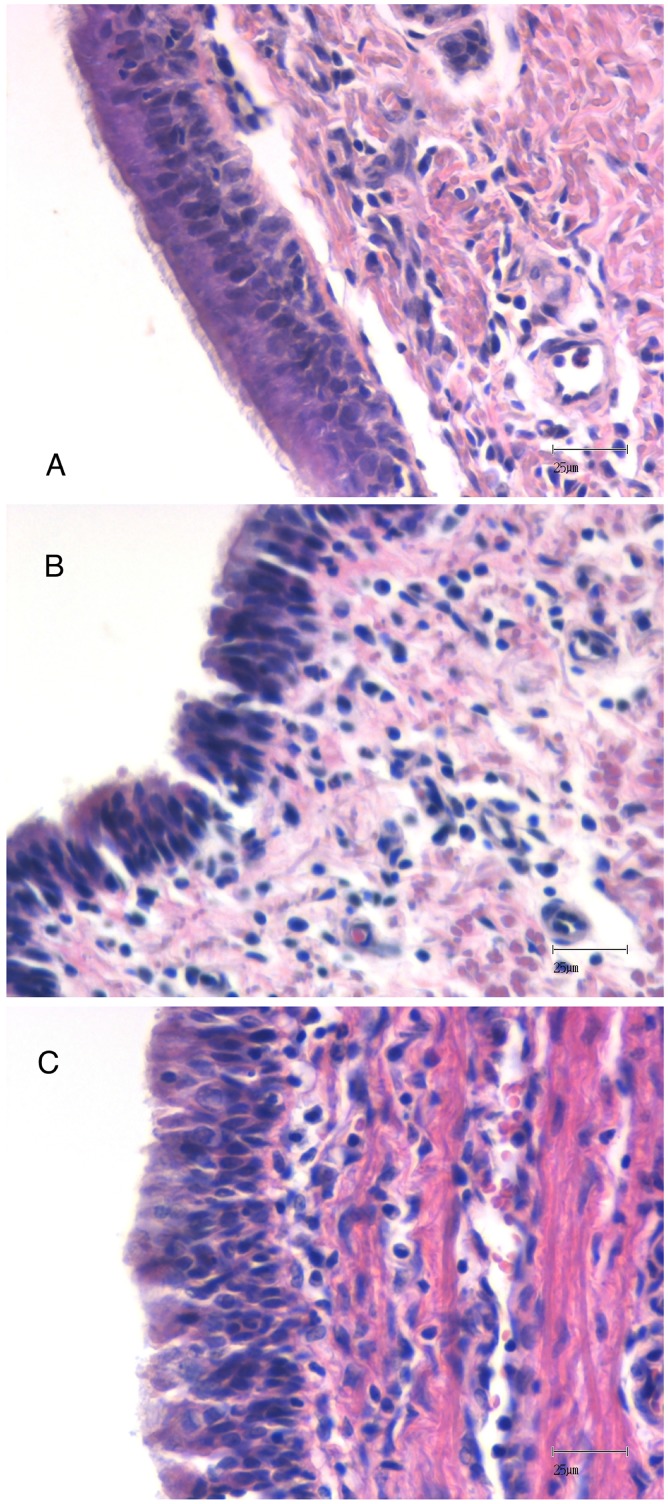
Representative photomicrographs stained with hematoxylin and eosin from tracheal epithelial tissue obtained at the end of the experiment in the control (panel A) and CPB groups (panels B and C). Panel A show the presence normal ciliated epithelium in the control group. On the other hand, panels B and C show regions of epithelial cilia loss and submucosal neutrophil infiltration in the CPB group.

Fig 3 shows representative photomicrographs of the epithelium of the control and CPB groups from tracheal samples obtained at T180. The ciliated epithelium of the control group was preserved, and a shortage of cilia in the epithelium of the CPB group was observed. We also observed increased neutrophil infiltration and submucosal edema in the CPB group.

## Discussion

Since its introduction in clinical practice, cardiopulmonary bypass performed during cardiac surgery has been associated with a respiratory dysfunction clinically characterized by a worsening in gas exchanges due to mechanical and inflammatory injuries to the respiratory system.[15–17] However, little is known about the acute effects of CPB on the function of the lower respiratory mucociliary system, which is the first line of defense of the airways against inhaled bacteria and particulate matter. In this study on the impact of CPB on the respiratory mucociliary system, we observed 1) a reduction in CBF at the end of study in the CPB group but not in the control group; 2) an increase in respiratory mucus viscosity at T90 in the CPB group compared to the control group; and 3) loss of tracheal epithelial cilia associated with submucosal edema and neutrophil infiltration as demonstrated by tracheal tissue histology.

Normal mucociliary system activity depends on an intact ciliated epithelium with synchronized ciliary beating, an adequate amount of mucus with ideal viscoelastic properties and the periciliary fluid layer, which may be compromised by mechanical and inflammatory injuries.[18] Exposure of blood elements to CPB circuit components induces a systemic inflammatory response that reaches maximal intensity 2 to 4 hours after CPB.[19, 20] In the lungs, this inflammatory reaction is further worsened by pulmonary ischemia caused by the interruption of pulmonary artery flow into the pulmonary capillaries, while the bronchial artery flow is negligible. According to Schlensak et al., the bronchial artery blood flow decreases from 4.8% to 1% of total circulatory flow during CPB, despite adequate perfusion pressure.[21] After pulmonary reperfusion, pro-inflammatory cytokines IL-6 and IL-8 increase in concentration in the pulmonary veins compared to systemic circulation[22], which triggers the endothelial cells to express adhesion molecules that can bind to circulating neutrophils. [23, 24] The sequestered and activated neutrophils provoke an increase in endothelial permeability with extravasation of fluid and plasma components and penetrate into the interstitium by moving between endothelial cell tight junctions and through the basement membrane. [22, 25] Within the lung tissue, the neutrophils release bioactive lipids, cytokines, oxygen metabolites, and enzymes, such as elastase and matrix metalloproteinases, that injure basement membranes, extracellular matrix and type I pneumocytes. [26, 27] The same inflammation-induced damage to cellular membranes and interstitium occurring in the lungs can injure the outer ciliary membrane continuous with the cell surface and ciliary apparatus that are projected deep into the cell cytoplasm.[28] These findings are in accord with our results.

Along with the CPB-induced inflammation of tracheal epithelial tissue, a reduction in bronchial artery blood flow during CPB leads to a reduction in energy metabolism and to a loss of effectiveness of high-metabolic-rate processes, such as epithelial ciliary beating and production of periciliary fluid.[21, 28] By taking together the reduction in ciliary beat frequency and the histological alterations observed in the tissue samples obtained after CPB, we can hypothesize that CPB contributed to the morpho-functional changes observed in tracheal epithelia in the experimental group by two main mechanisms: CPB-induced tracheal tissue inflammation and reduced tracheal epithelial perfusion during CPB.

Conditions other than the CPB could have been associated with the decrease in CBF in the CPB group, such as airway manipulation, artificial ventilation using elevated airway pressures, drugs and alterations in temperature and humidity conditions within the airways. In this study, the animals’ airways were manipulated on several occasions (tracheal intubation, tracheostomy, mucus sampling and tracheal fragment excision) and were exposed to high inspiratory oxygen concentrations and inhaled anesthetics, which can then lead to epithelial damage and decrease tracheal mucus velocity.[9, 13, 29–31] However, because the episodes of tracheal manipulation were similar in both groups and fragments of the trachea were collected from the same anatomical position at the same moment (90 minutes after the initiation of ventilation in the CPB group), the contribution of this mechanism to the reduction of CBF in the CPB group is negligible.

On the other hand, there were no differences in the *in situ* MCT measurements between groups or between times within either group. The fluid used to evaluate *in situ* MCT had physicochemical characteristics different from mucus and closer to those of water, which decreased the work performed by the epithelium. The distance over which *in situ* MCT was measured was short, which decreased the sensitivity of the test. Finally, there was great variability among all the samples, leading to a decrease in statistical power to detect a true difference.

To adequately simulate the overall effects of cardiac surgery on mucociliary system activity, mechanical ventilation was interrupted when CPB and the respiratory circuit disconnected. [32] At T90, immediately after the end of CPB, the apparent mucus viscosity was greater in the CPB group compared to the control group. This result may represent a dehydration of the tracheal respiratory mucus caused by a decrease in the airway water vapor concentration transported by inhaled gases during the ventilation. We cannot exclude the possibility that the interruption of ventilation and disconnection of the tracheal tube from the respiratory circuit during full mechanical circulation support, as performed during clinical practice, contributed to the decrease in CBF observed in the moment after CPB. However, considering that temporary interruption of mechanical ventilation does not induce mechanical or inflammatory injuries in tracheal epithelia and the viscosity of respiratory mucus in the CPB group was restored to baseline conditions of humidified mechanical ventilation during the 90 minutes after the end of CPB, we can assume that the role of mechanical ventilation interruption on mucociliary function was minimal and was reversed upon restarting ventilation.

This study has some limitations. We did not investigate the influence of CPB on tracheal periciliary fluid regulation. Because the periciliary fluid is produced by active control of ionic gradients across ciliated cell membranes and protein secretion by ciliated cells, it is possible that a derangement of this component of the mucociliary transport, once epithelia was injured after CPB, also contributed to our results. It is also of note that the CPB priming included mannitol solution that transiently reduces blood viscosity [33] and may reduce the viscosity of mucociliary fluid when inhaled by cystic fibrosis patients. [34] Therefore, the use of mannitol may create potential bias. However, we did not study the individual roles of ischemia of the pulmonary circulation induced by aortic cross-clamping, individual components of the CPB priming solution and CPB-induced SIRS. To investigate these, it would be necessary to perform separate pulmonary and systemic extracorporeal circulation experiments, which is beyond the scope of this study and is a subject for another investigation. Another point to be discussed is that heparin and protamine administration can trigger immunological responses that ultimately can alter vascular permeability and contribute to the findings of the study. Nevertheless, from the clinical point of view, the animals did not present any signs of reaction to either heparin or protamine infusion. Although we can’t individualize their possible contribution to the results of the study, we can assume that any contribution of heparin and protamine administration is small, since it did not induce any other systemic effects. Furthermore, in clinical practice, the infusion of heparin and protamine is an integral part of any CPB installation and weaning. Therefore, any possible contribution of these drugs to the findings must be accepted as a part of the CPB effect.

Finally, although no statistical differences were observed in the serial hematocrit measurements between the groups, there was a trend towards lower values in the CPB group. After starting the CPB, the circuit prime volume may have induced some degree of hemodilution. Unfortunately, we don’t have data on how low the hematocrit may have fallen. However, at the end of CPB, the mean hematocrit of CPB group had decrease from 28.4% at baseline to 26.5%. Since we kept the prime volume within the circuit at the end of the bypass and we did not performed hemofiltration, we believe that this little decrease in hematocrit is representative of the hemodilution occurring during CPB and indicates that it was minimal.

In conclusion, we observed the presence of inflammation and loss of cilia in tracheal epithelia, accompanied by a slight reduction in CBF and a transient increase in mucus viscosity following experimental CPB. It remains to be tested whether the same phenomena occur in patients undergoing CPB during cardiac surgery and if these alterations in the mucociliary system bear any clinical implications.

## Supporting Information

S1 File Hemodynamic and ventilatory data tables in Microsoft Excel format. (XLS)

S2 File Ciliary beat frequency data table in Microsoft Excel format. (XLSX)

S3 File Mucus viscosity data table.xls. (XLS)
